# Prospects on the Potential In Vitro Regenerative Features of Mechanically Treated-Adipose Tissue for Osteoarthritis Care

**DOI:** 10.1007/s12015-020-10099-2

**Published:** 2021-01-19

**Authors:** G. Desando, I. Bartolotti, L. Cattini, M. Tschon, L. Martini, M. Fini, A. Schiavinato, C. Soranzo, B. Grigolo

**Affiliations:** 1 Laboratorio RAMSES, IRCCS Istituto Ortopedico Rizzoli, Bologna, 40136 Italy; 2 Laboratorio di ImmunoReumatologia e Rigenerazione Tissutale, IRCCS Istituto Ortopedico Rizzoli, Bologna, 40136 Italia; 3 Scienze e Tecnologie Chirurgiche, IRCCS Istituto Ortopedico Rizzoli, Bologna, 40136 Italia; 4 Fidia Farmaceutici S.p.A, Abano Terme, Padova, 35031 Italy

**Keywords:** Osteoarthritis, Adipose stromal vascular fraction, Adipose-derived stromal cells, Mechanical technique, Enzymatic treatment, Cell characterization, Cartilage repair, Bone repair

## Abstract

Gathering a better grasp on the adipose stromal vascular fraction (SVF) is demanding among clinicians for osteoarthritis (OA) care because of its promising but multifaceted clinical outcomes. The aim of this preclinical in vitro study was to test whether the mechanical approach with Hy-Tissue SVF system, a class IIa CE marked device of adipose tissue micro-fragmentation, influences the biological features and functions of SVF. We compared mechanical generated-SVF (mSVF) with the enzymatic generated-SVF (eSVF) by testing cell survival, phenotype, differentiation, and paracrine properties using ELISA assays. Both adipose SVF showed 80% viable cells and enrichment for CD-44 marker. The mSVF product preserved the functions of cell populations within the adipose tissue; however, it displayed lowered nucleated cell recovery and CFU-F than eSVF. As for multipotency, mSVF and eSVF showed similar differentiation commitment for osteochondral lineages. Both adipose SVF exhibited an increased release of VEGF, HGF, IGF-1 and PDGF-bb, involved in pathways mediating osteochondral repair and cell migration. Both mSVF and eSVF also displayed high release for the anti-inflammatory cytokine IL-10. After in vitro culture, supernatants from both mSVF and eSVF groups showed a low release of cytokines except for IL-10, thereby giving evidence of functional changes after culture expansion. In this study, mSVF showed active cell populations in the adipose tissue comparable to eSVF with excellent survival, differentiation and paracrine properties under a new mechanical adipose tissue micro-fragmentation system; thereby suggesting its potential use as a minimally invasive technique for OA treatment.

**Supplementary Information:**

The online version contains supplementary material available at 10.1007/s12015-020-10099-2.

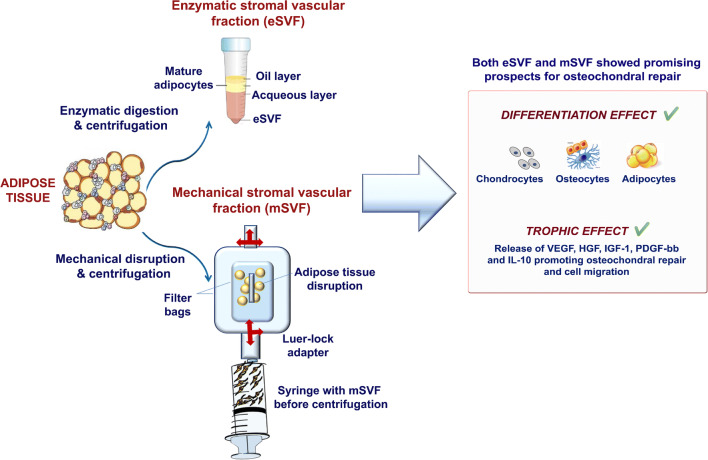

## Introduction

Osteoarthritis (OA) is one of the most common musculoskeletal disorders, characterized by osteochondral damages and synovial inflammation with a high cost for the healthcare system [1, 2]. Although many treatments for OA care exist, prosthesis represents the only option for patients with end-stage OA; therefore, posing the challenge for identifying innovative strategies to this unmet clinical need [3]. Among regenerative approaches, cell-based therapy is an excellent promise in delaying invasive surgical treatments with a considerable success rate [4–7]. In the last decade, better knowledge of the regenerative potential of mesenchymal stromal cells (MSCs), originated from several tissue sources, contributed to boosting their clinical translation [8–12]. In particular, adipose-derived mesenchymal stromal cells (ASCs) emerged as ideal candidates for OA care because they display a higher cell density and immunomodulatory potential than bone marrow-derived MSCs [13–16]. Many experts have examined the regenerative ability of cell populations within the adipose tissue, known as the adipose stromal vascular fraction (SVF). SVF is a supply of heterogeneous cell populations where ASCs, progenitor cells, pericytes, endothelial and immune cells drive repair processes via differentiation and paracrine activities [4, 14, 15]. ASCs can synergically work with immune cells to inhibit inflammatory and catabolic responses [17–21]. Achieving adipose SVF can envisage the use of both enzymatic and non-enzymatic methods; however, the lack of standardized protocols prevents clear-cut results on their performance [22, 23]. Thanks to technological advances, new research perspectives have opened in this field with the generation of point-of-care (POC) systems [24–26].

These devices work in a closed apparatus and ensure the least manipulation of autologous biological samples through a one-step procedure. The European Medicines Agency (EMA) does not recognize these samples as advanced therapy medicinal products, thereby facilitating their use in the clinical setting [27, 28]. Enzymatic systems can be up to 1000 times more efficient in cell recovery than the mechanical ones [22]. After enzymatic methods, the yield of nucleated cells (NC) ranges from 1 × 10^5^ to 1.3 × 10^6^ per ml of lipoaspirate. After the mechanical procedure, there is instead a range of NC from 1 × 10^4^ to 2.4 × 10^5^ per ml of lipoaspirate [22]. Both mechanical and enzymatic techniques for isolating adipose SVF do not require cell expansion, thereby limiting related issues and expensive surgical procedures.

Further preclinical studies are necessary to identify suitable POC systems for processing and preserving the biological features of SVF. Selecting the proper method is required to examine several aspects, including cellular survival, cell yield, presence of GMP facilities and, last but not least, the disease severity [24–26]. The main goal of this study was to assess in vitro the impact of the mechanical technique with Hy-Tissue SVF system, a class IIa CE marked device of adipose tissue micro-fragmentation, on the biological features and physiological functions of the generated adipose product (mSVF) with a direct comparison with the enzymatic SVF (eSVF). We also assessed the cytokine profile of eSVF and mSVF in the basal condition and following in vitro culture via ELISA assays. Research design envisaged tests on cell viability, multipotency and paracrine properties. Results from this study provided preliminary in vitro insights on several regenerative characteristics of mSVF, produced by a new mechanical adipose tissue micro-fragmentation, similar to eSVF, which could be suitable for tissue repair in OA.

## Materials and Methods

### Adipose Tissue Processing and Cell Viability Assessment of mSVF and eSVF

By implementing the 3Rs principles to reduce animal experimentation, tissues were retrieved from animals belonging to other unrelated and authorized research protocols not involving adipose tissue. Under general anaesthesia, adipose tissue was harvested under aseptic conditions from the inguinal fat pad of six adult male New Zealand White outbred rabbits (age: 26 weeks, Charles River Laboratories Italia S.R.L). We divided twenty grams of lipoaspirate (LA) into two parts and processed either by mechanical dissociation with the Hy-Tissue SVF system (Fidia Farmaceutici, Abano, Italy) or only with the enzymatic treatment. The Hy-Tissue SVF system allowed mechanical disruption of LA for each sample by producing the mSVF. Briefly, ten grams of LA was finely minced and loaded with 30 ml of saline in a closed sterile bag for mechanical fragmentation by manual massage for 8–10 min at room temperature (RT). Disrupted LA was recovered through a 120 μm filter and transferred via a Luer adapter into a syringe. The ensuing step foresaw cell separation from the disrupted LA through centrifugation at 400 g for 10 min, with a cell concentration device (Duografter II Fidia Pharma Group Company). We obtained about 6 ml of mSVF in a processing time of 30 min. We discarded floating oil and tissue debris (in-between/intermediate layer) and transferred the cell pellet and the aqueous supernatant in a tube for preclinical in vitro examinations. For eSVF preparation, the remaining ten grams of LA were enzymatically-digested with collagenase NB4 (Serva Electrophoresis, GmbH, Heidelberg, Germany) as reported in our previous study [19]. Microscopic evaluations (Nikon, Tokyo, Japan) and Live and Dead test (Thermo Fisher Scientific, Waltham, MA, US) tested cell yield, morphology and viability on eSVF and mSVF, as listed in our past work [19].

### Phenotypic Characterization of ASCs from mSVF and eSVF

We analyzed the immunophenotype of ASCs got from mSVF and eSVF with BD FACS Canto II flow cytometer (Becton Dickinson, New Jersey, US). We analysed several hematopoietic, non-hematopoietic and mesenchymal markers using the following unlabelled mouse anti-rabbit primary antibodies: CD-44 (1 μg/ml) (Origene, Maryland, US), CD-90 (2 μg/ml) (Thermo Scientific, Massachusetts, US), CD-105 (5 μg/ml) (GeneTex, California, US), CD-146 (1 μg/ml) (Novus, Missouri, US), CD-13 (4 μg/ml) (GeneTex, CA, US), CD-14 (2.5 μg/ml) (Origene), CD-31 (0.5 μg/ml) (Origene), CD-45 (2 μg/ml; Bio-Rad-AbD Serotec, Oxford, UK) and CD-68 (5 μg/ml) (Biorbyt, Cambridge, UK). We then diluted and incubated samples in phosphate-buffered saline (PBS) enriched with 0.2% sodium azide (NaN_3_) (Sigma-Aldrich, Missouri, US) and 2% Fetal Bovine Serum (FBS) for 30 min at 4 °C. Afterwards, specific negative and isotype controls were carried out for IgG1 and IgG2a. After the PBS washing steps, we incubated biological samples with the goat anti-mouse secondary antibody conjugated to fluorescein isothiocyanate (FITC) (5 μg/ml) (Thermo Scientific). Finally, biological samples were acquired and analysed with BD FACS CANTO II flow cytometer. Forward versus side-scatter (FSC vs SSC) gating was used for identifying the cell population of interest and excluding debris. The isotype controls were used to determine the level of non-specific binding by establishing a threshold of positivity for each marker (See supplementary Fig. 1 and Fig. 2).

### Investigation of Multipotency of ASCs from mSVF and eSVF

Fibroblast colony-forming units (CFU-F) and the differentiation potential were tested to assess the multipotency of mSVF (*n* = 6), and eSVF (*n* = 6). Firstly, we counted nucleated cells in eSVF and mSVF groups with Turk dye and then plated 2 × 10^4^ nucleated cells for each group at passage p0 in 56 cm^2^ Petri dishes (Corning Lifesciences, Tewksbury, MA) to perform CFU-F test at day 14. Cell duplicates were cultured with complete alpha minimum essential media (α MEM) (Sigma-Aldrich) enriched with 100 U/ml penicillin G (Sigma), 50 μg/ml ascorbic acid (Sigma) and 15% Foetal Bovine Serum (FBS) at 37 °C and 5% CO_2_ in the incubator (Thermo Scientific). Colonies were stained with 2% crystal violet solution (Sigma-Aldrich), and colonies with more than 50 cells were counted at the inverted microscope (Nikon). After seeding ASCs from eSVF and mSVF groups at passage 0 in 12 well plates, cells were then cultured with osteogenic, adipogenic and chondrogenic differentiation media with two changes per week. As for osteogenesis, we used α-MEM supplemented with 100 U/ml penicillin G (Sigma), 100 μM ascorbic acid (Sigma), 100 nM dexamethasone (Sigma), 10 mM β glycerol phosphate (Sigma) and 15% FBS. Regarding adipogenesis, D-MEM High Glucose (ThermoFisher Scientific) supplemented with 500 μM 3-isobutyl-1-methylxanthine (IBMX) (Sigma), 100 μM indomethacin (Sigma), 10 μM insulin (Sigma), 1 μM dexamethasone (Sigma) and 15% FBS were used. Regarding chondrogenic differentiation, ASCs were expanded from eSVF and mSVF in the complete culture medium to achieve the desired cell number for preparing pellet cultures. Moreover, 3D pellet cultures were set up at passage 2 and then cultured with high glucose Dulbecco’s-MEM containing 10^−7^ M dexamethasone (Sigma), 50 μg/ml ascorbate-2-phosphate (Sigma), 1 mM sodium-pyruvate (Sigma), 100× ITS premix (BD Biosciences, San Jose, CA, US) and 10 ng/ml TGFβ3 (Miltenyi Biotec, Bologna, Italy). Assessments on osteogenesis and adipogenesis were carried out on day 0, 14 and 21 with Alizarin Red (ARS) and Oil Red. Biological duplicates stained with Oil Red solution were firstly pre-treated with 60% isopropanol for 20 min at RT before being fixed with buffered formalin (Kaltek, Padova, Italy). Following these steps, samples were stained with ARS and 0.5% Oil Red (Sigma) at the established experimental times. Osteogenesis and adipogenesis were quantified by reading the absorbance at 510 and 517 nm, respectively. A spectrophotometric analysis with TECAN Infinite® 200 PRO (Tecan Italia, S.r.l., Cernusco Sul Naviglio, Italy) was performed to assess mineral apposition and lipid droplets formation. We used the wavelength of 510 nm for quantifying ARS and 517 nm for Oil Red staining, which we defined through a pilot scan of wavelengths (350–750 nm) in a limited number of samples. The “multiple reads per well” (15 × 15 size, circle filled pattern; 177 captured fields/well) was carried out to ensure a good representation of the whole well’s surface. As for chondrogenesis, we fixed pellet cultures with buffered formalin (Sigma) at days 0 and 28, washed with PBS and later frozen in liquid nitrogen and kept at −80 °C. We cut sections at the cryostat and stained with 0.1% Safranin-O/0.02% Fast Green (Sigma) to test proteoglycan/collagen composition. We examined pellet cultures with the Bern score system, which analyzes the uniformity and intensity of safranin staining, formed matrix, and cellular architecture. The system score ranges from 0 to 9, where 0 points indicates chondrogenesis, whereas 9 points to the highest chondrogenesis [29]. Immunohistochemical analysis was performed for type I (2 μg/ml) (Sigma) and type II (2 μg/ml) (II-II6B3, Hybridoma Bank, University of Iowa, US) collagens to evaluate chondrogenic differentiation [19]. We assessed the percentage of positivity by defining a threshold with the Hue Saturation Intensity (HIS) system with the NIS-Elements software and Eclipse 90i microscope (Nikon). We adopted a scale indicating no positive signals with 0% and the highest protein expression with 100%.

### Enzyme-Linked Immunosorbent Assay: Analysis of Released Cytokines in mSVF and eSVF

We performed evaluations on soluble factors from supernatants of uncultured mSVF and eSVF through the commercially enzyme-linked immunosorbent tests (ELISA). To this end, mSVF and eSVF were centrifuged at 600 g for 10 min, and their supernatants were collected to test the released cytokines. Moreover, we plated and cultured mSVF and eSVF to test whether the in vitro culture can modify their cytokine profile compared to the native uncultured adipose products. After 80% confluence, culture supernatants (S/N) from both mSVF (mSVF S/N) and eSVF (eSVF S/N) were recovered and kept in serum-free medium for 48 h before their collection. We performed ELISA assessments on the following molecules: vascular endothelial growth factor (VEGF) (Cloud-Clone Corp, Texas, US), Platelet-derived growth factor (PDGF)-bb (LifeSpan Biosciences, Washington, US), insulin growth factor (IGF)-1 (Cloud-Clone Corp), hepatocyte growth factor (HGF) (Cloud-Clone Corp), interleukin (IL)-10 (Cloud-Clone Corp), interleukin (IL)-1β (Cloud-Clone Corp), IL-6 (Cloud-Clone Corp), IL-21 (MyBioSource, California, US), IL-23 (MyBioSource), tumour necrosis factor (TNF)-α (Cusabio, Houston, Texas, US). We used the manufacturer’s instructions through spectrophotometer measurement at a wavelength of 510–570 nm.

### Statistical Analysis

GraphPad Prism6 software was used for statistical analysis. We performed data distribution using the Kolmogorov–Smirnov (K-S) test. The non-parametric Wilcoxon matched-pairs signed-rank test was used to test differences between eSVF and mSVF groups. Data were considered significant with *P* < 0.05. Data are presented as boxplots reporting Median (Mdn), lower and upper quartiles, minimum-maximum (Min-Max) range and outlier values.

## Results

### mSVF and eSVF Showed a High Percentage of Cellular Viability

**Fig. 1:**
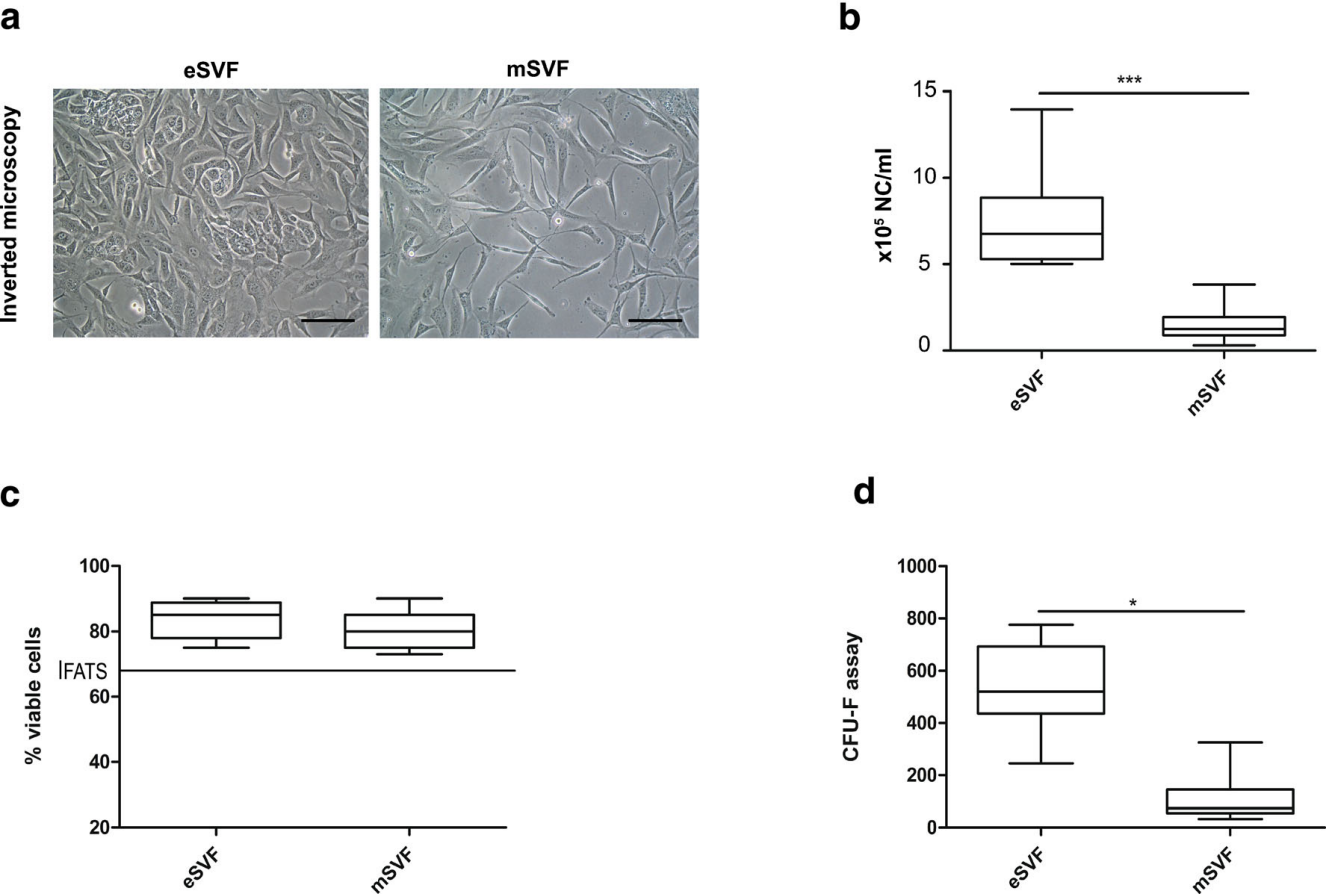
**a** Representative images of inverted microscopy of ASCs from mSVF and eSVF, scale bar: 100 μm. **b-d** The panels show Tukey box plots of the number of multinucleated cells (NC) isolated per ml (**b**); the percentage of viable cells (**c**); the number of CFU-Fs per ml in eSVF and mSVF at day 14 (**d**). The dashed line shows the threshold of 70% provided by the International Federation for Adipose Therapeutics and Science (IFATS) [30]. Results from Wilcoxon matched-pairs signed-rank test are reported (*n* = 6 paired samples each). ^***^P < 0.001 NC in eSVF versus NC in mSVF; ^*^P < 0.05 CFU-Fs in eSVF versus mSVF

**Fig. 2:**
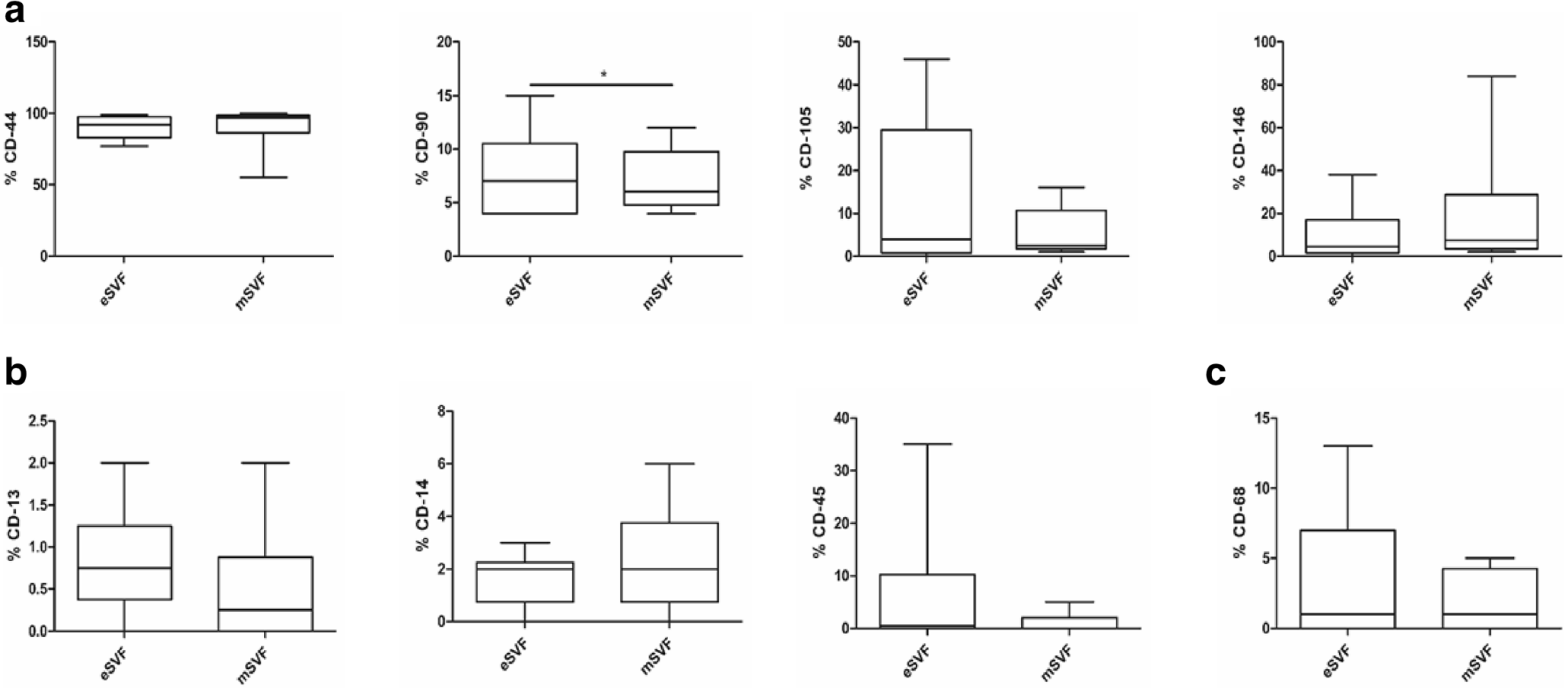
**a** The panels show Tukey box plots of the percentages of the mesenchymal markers: CD-44, CD-90, CD-105 and CD-146 in ASCs from eSVF and mSVF. **b** Tukey boxplots of the rate of the hematopoietic markers: CD-13, CD-14, CD-45. **c**. Tukey boxplots of the percentage of the inflammatory macrophage marker, CD-68. Results of Wilcoxon matched-pairs signed-rank test are reported (*n* = 6 paired samples each). ^*^P < 0.05 % CD90 in eSVF versus mSVF

mSVF (*n* = 6 cases; median 1.3 × 10^5^; min-max 3 × 10^4^–3.8 × 10^5^) exhibited approximately five times lower yield of nucleated cells/ml per gram of adipose tissue when compared to eSVF (*n* = 6 cases; median 6.7 × 10^5^; min-max 5 × 10^5^–1.4 × 10^6^), following the enzymatic digestion (*P* < 0.0001) (Fig. 1a-b). Microscopic assessments of eSVF and mSVF, plated on culture flasks on day 14, showed a heterogeneous population with elongated and polygonal cell morphologies, and lipid droplets in their cytoplasm. Regardless of adipose tissue processing, ASCs displayed a high viability rate in both eSVF (median 85%; min-max 75–90%) and mSVF (median 80%, min-max 73–90%), as documented by Live and Dead assay (Fig. 1c). CFU-F analysis gave evidence that ASCs from eSVF (*n* = 6) (median 520; min-max 245–775) formed on average 7 times more CFU-Fs per ml of lipoaspirate than mSVF (*n* = 6) (median 73.75; min-max 32.50–325) (*P* < 0.05) (Fig. 1d). A few differences between eSVF and mSVF was noticed after flow cytometry. About mesenchymal markers, eSVF and mSVF exhibited similar high expression for CD-44 marker. In contrast, mSVF showed slightly higher levels of CD-146 when compared to eSVF. mSVF showed lower levels for CD-105 and CD-90 than eSVF, with significant results only for CD-90 (*P* < 0.05) (Fig. 2a). Both groups displayed a low expression for the hematopoietic markers CD-13, CD-14 and CD-45 (Fig. 2b) and the inflammatory macrophage marker CD-68 (Fig. 2c).

### eSVF and mSVF Showed Similar Differentiation Potential towards the Osteochondral Lineage

**Fig. 3:**
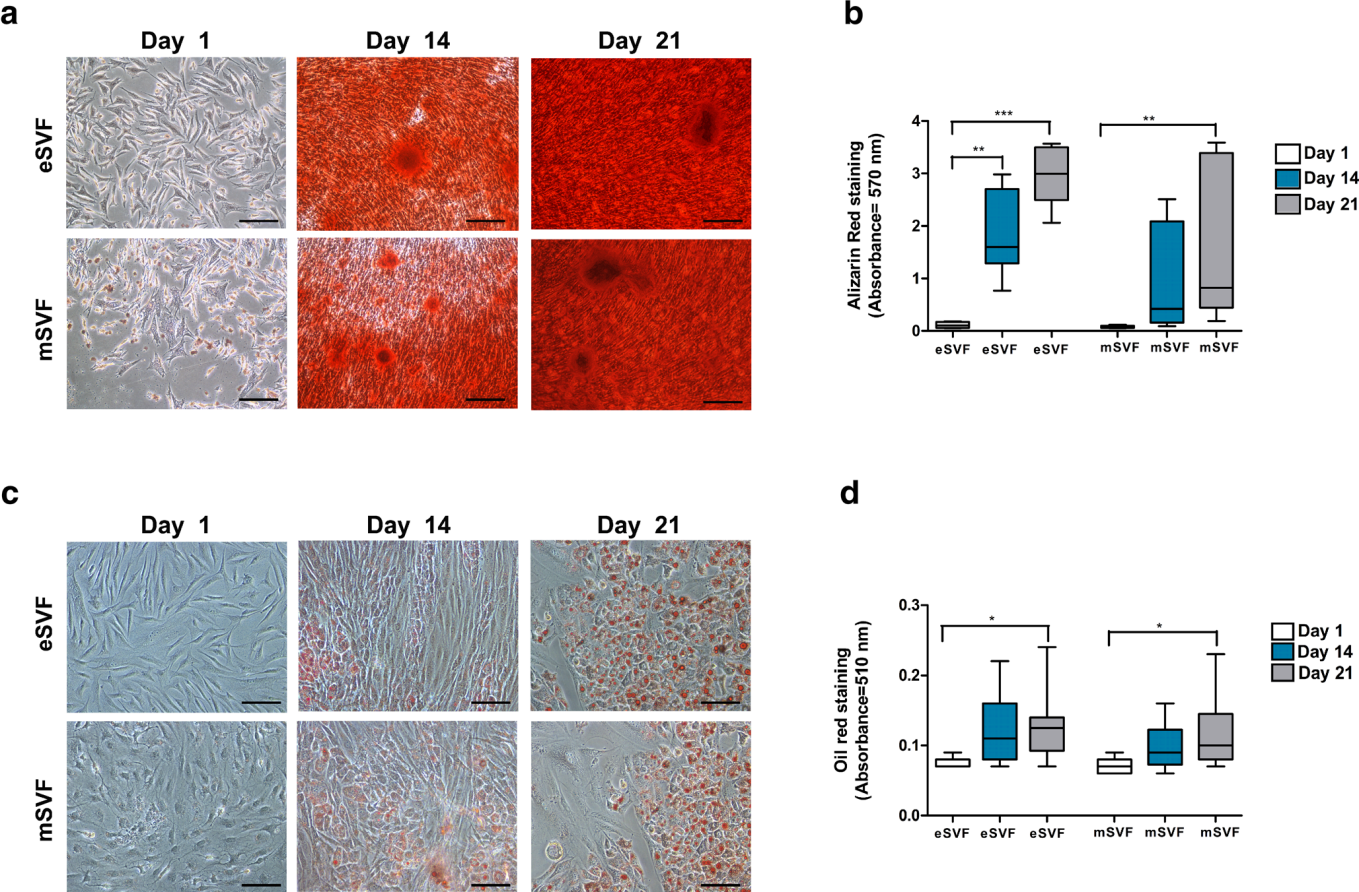
**a** Representative micrographs of ASCs from eSVF and mSVF stained with Alizarin Red after the culture with the osteogenic medium on day 1, 14 and 21. Red staining: mineralizing regions. Scale bar: 100 μm. **b** The Tukey box plots of osteogenesis reported as absorbance values at 517 nm in eSVF and mSVF. Results of the Wilcoxon matched-pairs signed-rank are reported (*n* = 6 paired samples each). ^**^P < 0.01 eSVF group at day one versus day 14; ^***^P < 0.001 eSVF group at day one versus day 21; ^**^P < 0.01 mSVF group at day one versus day 21. **c** Representative micrographs of ASCs from eSVF and mSVF stained with Oil Red after the culture with the adipogenic medium at 1, 14 and 21 days. Red staining: intra-cytoplasmatic lipid regions. Scale bar: 100 μm. **d** the Tukey boxplots of adipogenesis reported as absorbance values at 510 nm in eSVF and mSVF. Results of the Wilcoxon matched-pairs signed-rank are reported (*n* = 6 paired samples each). ^*^P < 0.05 eSVF group at day one versus day 21; *P < 0.05 mSVF at day one versus day 21

**Fig. 4:**
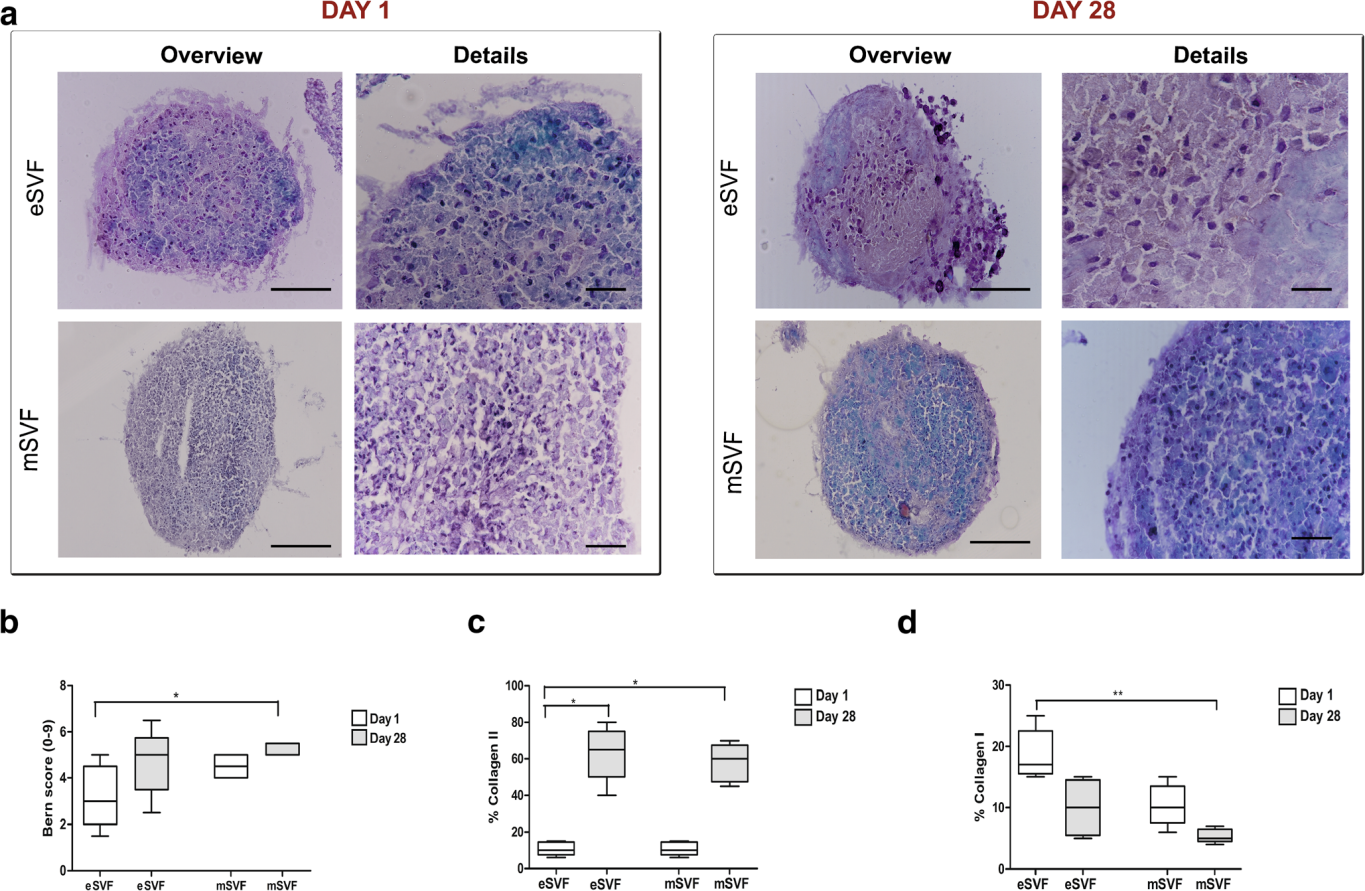
**a** Representative micrographs of pellet cultures of ASCs from eSVF and mSVF stained with Safranin-O/Fast Green staining at 1 and 28 days. Pinkish staining: slight proteoglycan content. Scale bar: 100 μm. **b**-**d** The Tukey box plots of chondrogenesis measured with Bern score (**b**), percentage of positivity for collagen II (**c**) and collagen I (**d**) in eSVF and mSVF. Results of the Wilcoxon matched-pairs signed-rank are reported (*n* = 6 paired samples each). ^*^P < 0.05 Bern score in eSVF on day one versus mSVF on day 28; ^*^P < 0.05 % collagen II in eSVF on day one versus eSVF on day 28; ^*^P < 0.05 % collagen II in eSVF on day one versus mSVF on day 28; ^**^P < 0.01 % collagen I in eSVF on day one versus mSVF on day 21

ASCs from eSVF (*n* = 6) and mSVF (*n* = 6) showed similar osteogenic (Fig. 3a-b), adipogenic (Fig. 3c-d) and chondrogenic (Fig. 4a-d) differentiation. Regarding osteogenesis, eSVF and mSVF showed best results at day 21. Both groups promoted stronger mineral apposition on day 21 than day one (*P* < 0.001 and *P* < 0.01), with a more regular distribution in the eSVF (Fig. 3a-b). As for adipogenesis, both eSVF and mSVF showed high lipid-droplets formation at day 21. eSVF gave evidence of a higher presence of intra-cytoplasmic lipids in ASCs at day 21 than day one (*P* < 0.05) (Fig. 3b-c). Likewise, mSVF displayed a stronger adipogenesis potential at day 21 rather than day one (*P* < 0.05) (Fig. 3 c-d). eSVF and mSVF (median 5; min-max 5–5.5) displayed similar Bern score on day 28, showing moderate chondrogenic processes. On day 28, pellet culture sections in eSVF and mSVF showed moderate staining for Safranin-O, moderate cell density with mixed spindle/fibrous and rounded chondrogenic morphology. eSVF presented lower Bern score on starting time than mSVF on day 28 (*P* < 0.05) (Fig. 4a-b). Investigating the sub-criteria of Bern score showed higher safranin-O and matrix formation in mSVF than eSVF on day one, but with no statistical evidence. Semi-quantitative analysis on type II collagen gave evidence of lower protein expression in mSVF when compared to eSVF on day 28, but with no significant evidence (Fig. 4c). Conversely, mSVF showed lower protein expression of collagen type I, typical fibrous marker, when compared to eSVF; however, the difference is not significant when comparing the same time points (Fig. 4d).

### mSVF and eSVF Released Typical Cytokines Involved in Tissue Repair

**Fig. 5:**
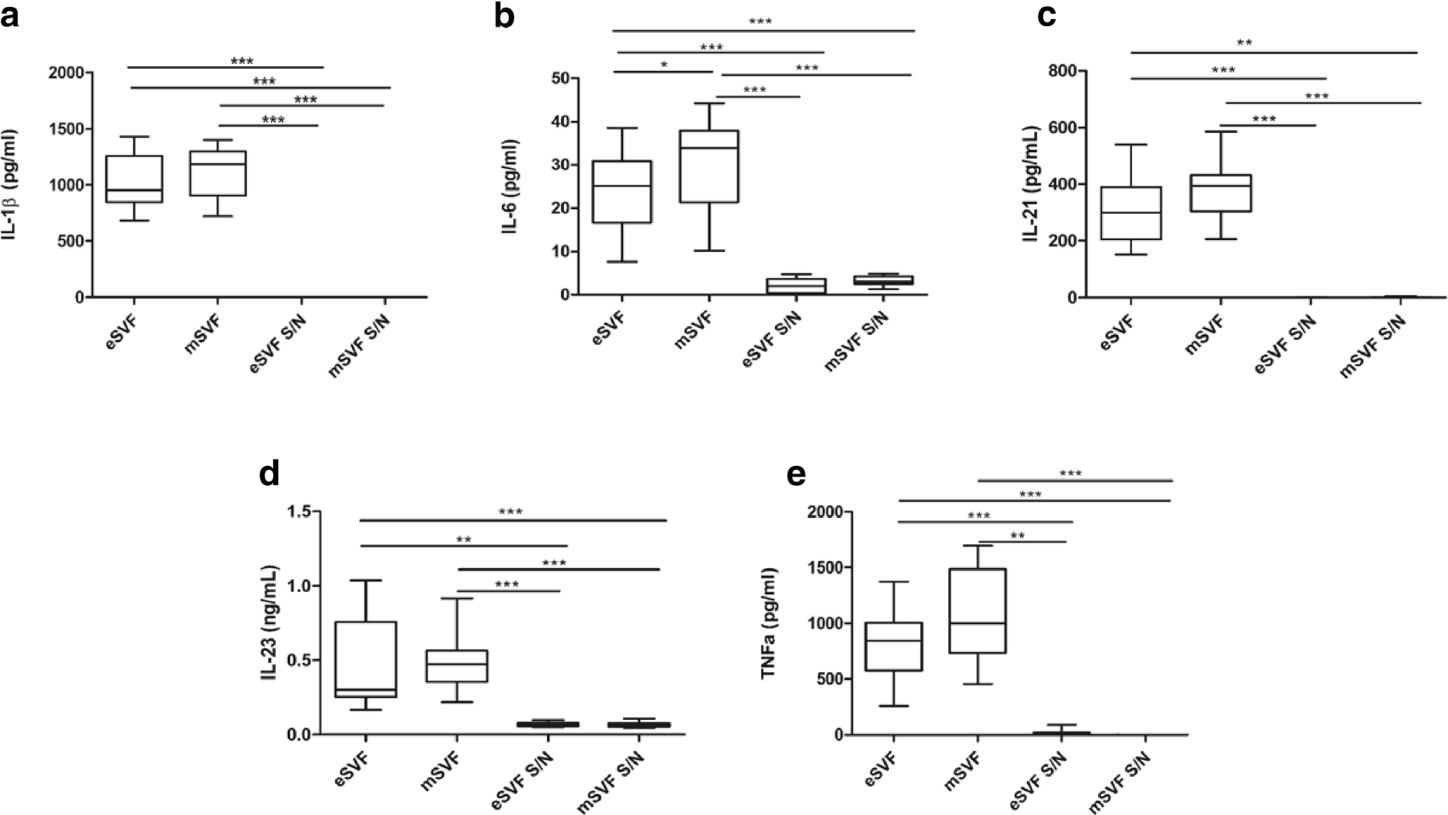
**a-d** The Tukey box plots show protein expression of interleukin (IL)-1β, IL-6, IL-21, IL-23 and tumour necrosis factor (TNF)-α in eSVF, mSVF and supernatants of cultured ASCs from eSVF (eSVF S/N) and mSVF (mSVF S/N), measured by ELISA assay. ^*^P < 0.05; ^**^P < 0.01; ^***^P < 0.01. Results of the Wilcoxon matched-pairs signed-rank are reported (*n* = 6 paired samples each)

**Fig. 6:**
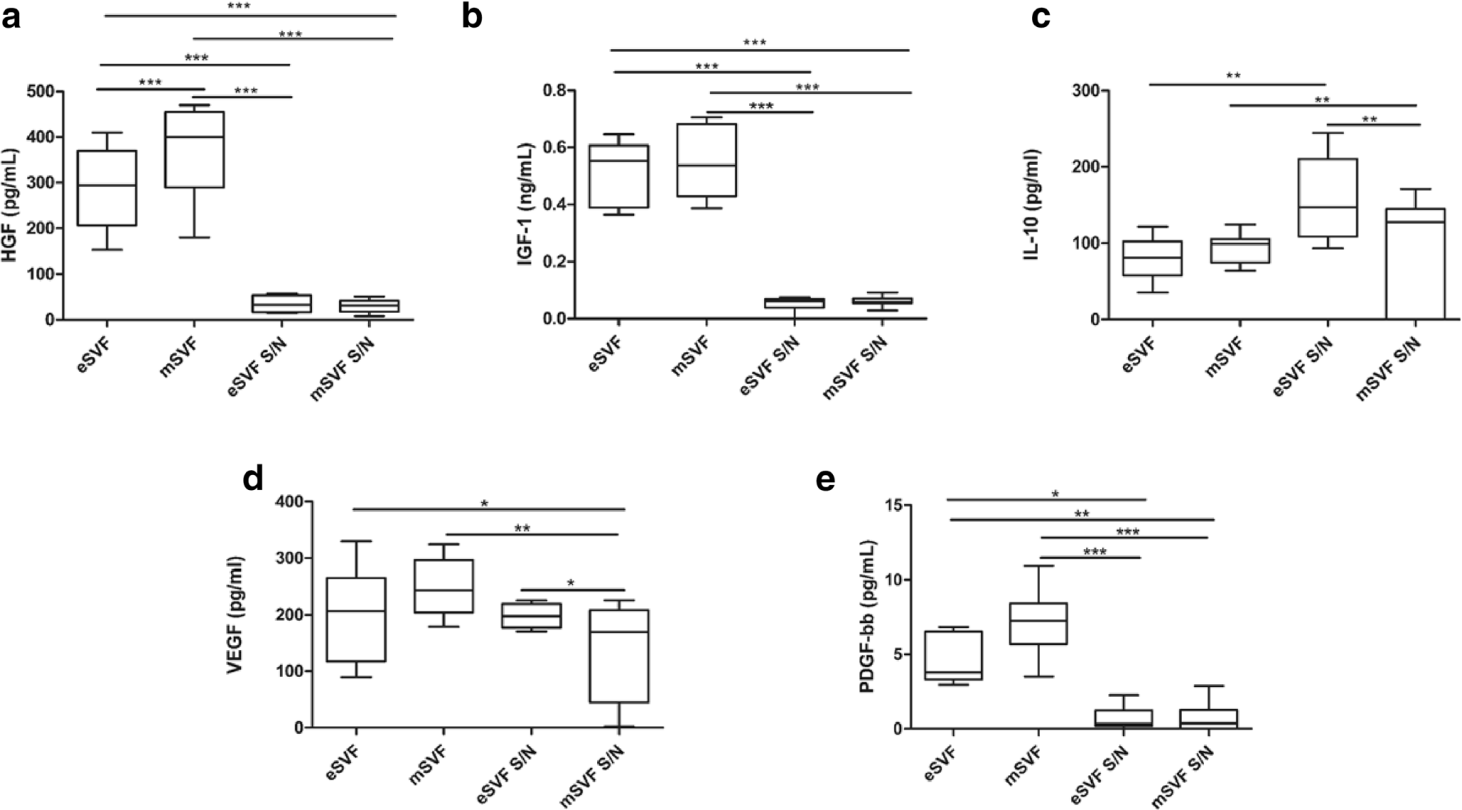
**a-d** The Tukey box plots show protein expression of Hepatocyte growth factor (HGF), Insulin Growth Factor (IGF)-1, interleukin (IL)-10, vascular endothelial growth factor (VEGF), platelet-derived growth factor (PDGF)-bb in eSVF, mSVF and supernatants of cultured ASCs from eSVF (eSVF S/N) and mSVF (mSVF S/N), measured by ELISA assay. ^*^P < 0.05; ^**^P < 0.01; ^***^P < 0.01. Results of the Wilcoxon matched-pairs signed-rank are reported (*n* = 6 paired samples each)

eSVF and mSVF released a high amount of the inflammatory cytokines IL-1β, IL-21 and TNF-α and moderate content of IL6 and IL-23. eSVF and mSVF presented higher cytokine release of these markers when compared to supernatants collected following the in vitro cultures of eSVF (eSVF S/N) and mSVF (mSVF S/N) (*P* < 0.05; *P* < 0.01; *P* < 0.001) (Fig. 5a-e). Differences between eSVF and mSVF groups were noticed only for the inflammatory mediator IL-6, where the eSVF showed the lowest value (*P* < 0.05) (Fig. 5b). Both eSVF and mSVF exhibited a high cytokine release of HGF, IL-10 and VEGF, and low values for IGF-1 and PDGF-bb (Fig. 6a-e). As for HGF, mSVF group showed higher cytokine release than eSVF (*P* < 0.001), eSVF S/N (*P* < 0.001) and mSVF S/N (*P* < 0.001) (Fig. 6a). As for IGF-1, both mSVF and eSVF groups displayed higher cytokine release than their respective supernatants (*P* < 0.001) (Fig. 6b). As for IL-10, eSVF S/N and mSVF S/N groups showed higher protein values than eSVF and mSVF (*P* < 0.01) (Fig. 6a-e). In particular, eSVF S/N showed higher IL-10 release than mSVF S/N (*P* < 0.01) (Fig. 6c). As for VEGF, eSVF S/N showed a higher cytokine release than mSVF S/N (*P* < 0.05) (Fig. 6d). Both mSVF and eSVF products showed a higher PDGF release than their supernatants (*P* < 0.001, *P* < 0.01, *P* < 0.05). In general, both eSVF and mSVF displayed higher release of HGF, IGF-1, VEGF and PDGF-bb than both eSVF S/N and mSVF S/N (Fig. 6a, b, d, e).

## Discussion

Adipose SVF-based therapy holds great promise for treating several musculoskeletal disorders, including OA [4, 17, 31]. Gathering better knowledge of techniques for isolating the adipose SVF is demanding among experts for reinforcing traditional systems for OA care [24, 26]. Biological insights on the healing potential of such approaches can be instrumental for clinicians to select the best therapeutic modality in handling various OA severity. The primary aim of this preclinical in vitro study was to test the impact of the mechanical treatment with the Hy-Tissue SVF system on the biological characteristics of the resulting adipose product, mSVF, with a direct comparison with eSVF. The main conclusions of this study showed similar biological features and functions of cell populations within the adipose tissue in mSVF and eSVF. Both adipose products showed good cell viability, differentiation commitment, and paracrine properties with important implications in supporting regenerative processes to counteract OA changes. The high cell viability of 80% recorded in both eSVF and mSVF can be considered as a promising aspect for their application in the clinical setting. Cell survival is the utmost clinical aspect, as non-viable cells may lead to severe inflammatory responses by altering tissue repair [22]. The presence of dead cells can trigger inflammatory responses via the recruitment of the immune host defence. Toll-like receptors (TLRs) from the immune system can recognize the “non self” or “hidden self” molecules released in the extracellular fluid from dead cells, with the ensuing release of inflammatory mediators [32]. In general, mSVF exhibited a lower recovery of nucleated cells than eSVF showing similar values to the results obtained with human LA processed with alternative mechanical techniques [22, 27, 31, 32] [30, 33]. The recorded small number is because of the less efficient cell release from LA after the only mechanical technique [34]. The mean value of the nucleated cells/ml per gram of LA obtained in our study with mSVF is, however, slightly higher of that reported in the majority of the studies on SVF using non-enzymatic methods [34]. Indeed, minimal requirements for defining the preclinical efficacy of an adipose treatment should include not only the number of nucleated, cells but also the phenotypic and functional features of cell populations in the processed adipose tissue. To this end, we first characterised eSVF and mSVF to provide first insights on their regenerative potential. Accordingly, we partially followed instructions set by the International Society for Cellular Therapy (ISCT) to give indications on the cell composition in mSVF [35]. It showed strong positivity for the surface marker CD-44, presenting a low presence for CD-13, CD-14 and CD-45, typical hematopoietic markers.

One limitation of this study is the analysis of a reduced panel of the CD for flow cytometry analysis due to the small availability of antibodies with rabbit reactivity and the lack of co-staining analysis. However, guidelines from ISCT show some shortcomings. In particular, there are no population-specific phenotypes as a vast range of cell types express common MSC markers, rendering complex a clear distinction [36]. To gain further insights on the multipotency, we investigated CFU-F and the differentiation potential of ASCs isolated from mSVF and eSVF. Data from CFU-F assay gave evidence that mSVF displayed seven times less CFU-Fs than eSVF, likely due to the high number of precursor cells in the latter. Despite the low number of CFU-F, ASCs from both eSVF and mSVF displayed a high differentiation commitment by giving first evidence on the benefit of using it to drive also osteochondral repair. Further preclinical in vivo studies are ongoing, however, essential to establish mSVF potential in OA microenvironment.

As reported here and in other similar works, the critical role of paracrine effects of ASCs on regenerative responses is quite evident, with enormous prospects for clinical treatments [37]. Along this line, this research tested and compared the cytokine release of mSVF and eSVF, selecting growth factors (GF) and cytokines involved in OA setting [1, 38–40]. Moreover, we compared mSVF and eSVF with their respective supernatants (S/N) to assess whether in vitro culture may alter the behaviour of such adipose products. mSVF displayed high protein levels of IL-1β, TNF-α and IL-21 with similar values to eSVF. The equal amount of such inflammatory mediators in eSVF and mSVF show that this expression did not depend on the micro fragmentation technology. Differently, from humans where liposuction by itself ensures adipose tissue fragmentation, rabbit adipose tissue, displaying more fibrotic features than humans, needed a preliminary manual shredding to obtain proper consistency for the micro-fragmentation with Hy-tissue SVF device. Both the mechanical fragmentation and the enzymatic treatment increased inflammatory markers. A reduced expression of CD-68, a typical marker of proinflammatory macrophages, was instead observed in both eSVF and mSVF. We can postulate that the highest release of inflammatory markers from both eSVF and mSVF could stem from the presence of a heterogeneous population, including immune cells.

Conversely, in vitro cell expansion likely favoured the selection of adherent cells; thereby, leading to a partial loss of immune cells in the supernatants samples. Notably, immune cells can have pleiotropic roles related not only to the host defence but also to tissue repair. Some evolutionarily conserved signalling pathways, such as MAPK-AP1 IKK-NF-κB, can also connect inflammatory stimuli to repair/regenerative processes [41]. New insights into the role of immune signalling gave evidence that inflammatory inputs from the immune cells can modulate the healing potential of mesenchymal stromal cells (MSCs) [42]. Notably, IL-1β, TNF-α and IL-21, highly expressed in both adipose products, can even play an essential part in mobilizing and stimulating critical regenerative players such as progenitor precursors and immune cells [43, 44]. Accordingly, several authors demonstrated that the priming with inflammatory cytokines can confer to MSCs a greater immunomodulatory potential and pro-trophic phenotype; thereby opening exciting insights on the potential use of preconditioning MSCs for future therapies [45, 46]. In this light, these properties would suggest the eSVF and mSVF potential in encouraging cell recruitment; even if further preclinical in vitro and in vivo studies are still necessary. Also, mSVF displayed a high release of IL-10, an anti-inflammatory cytokine produced by several immune cells, including Th2 cells, Tr1 cells and wound-healing macrophages, which exert several immunomodulatory effects [47]. Taking into account literature data reporting increased levels of bioactive molecules after adipose micro fragmentation, we examined a list of anabolic mediators [48]. We noticed the potential of both eSVF and mSVF in upholding some wound-healing responses. In particular, we found that both eSVF and mSVF exhibited an enhanced amount of VEGF and HGF, involved in regulating the angiogenesis processes for driving bone regeneration.

The pro-angiogenic effects can derive from the crosstalk between adipose-derived mesenchymal stromal cells (ASCs) and endothelial progenitor cells (EP) in the adipose tissue, as reported in various preclinical studies [49, 50]. Evaluation of such crosstalk showed the role of PDGF and the Notch signalling in regenerative medicine [51–53]. In this regard, PDGF-bb in mSVF and eSVF might support the crosstalk between ASCs and EP in driving tissue repair. HGF also stimulates proteoglycan synthesis and cell migration, thus, offering critical functions for cartilage growth and an outlook on the potential role of mSVF in this repair context [39]. The pronounced expression of IL-10 in eSVF and mSVF is another promising aspect with potential clinical significance, as this mediator regulates extracellular matrix formation and differentiation of EP [54, 55]. Data on IGF-1 and PDGF-bb gave further indications on the potential role of eSVF and mSVF in supporting cartilage repair. Both GFs suppress various responses mediated by IL-1β like NF-kB activation and chondrocyte apoptosis [40, 56]; thereby, supporting cartilage repair [56]. In this light, future preclinical studies, especially on mSVF as a source of GF, might be helpful to explain its therapeutic implications in OA setting [56, 57]. S/N from mSVF and eSVF exhibited protein reduction for all markers except for IL-10. We can speculate that the in vitro culture altered the interactions of cell-cell junctions and the ECM proteins in the extracellular environment, leading to substantial changes in cell morphology and functions. These findings underline how cells are so sensitive to their in vitro culture microenvironment in agreement with the literature [58]. Indeed, limiting cell manipulation and promoting one-step approaches as occurs in eSVF and mSVF, would prevent the need for GMPs facilities and costly surgical procedures. In summary, these results show the healing potential of mSVF as a minimum invasive method to tackle some OA issues, as it preserves the biological characteristics of cell populations within the adipose cell tissue by ensuring an optimal rate of cell viability and a paracrine behaviour similarly to eSVF.

## Conclusions

Our results give preliminary insights on the regenerative features of mSVF generated by the Hy-Tissue SVF device, a mechanical adipose tissue micro-fragmentation system, and compared with eSVF. The differentiation potential and bioactive molecules of both eSVF and mSVF offer exciting prospects on their regenerative potential for the treatment of osteochondral pathologies. The different cytokine release found in supernatants after culturing mSVF and eSVF than their original adipose products highlights the benefit in favouring one-step approaches by overcoming issues from in vitro cell manipulation. However, we underline the need for more in-depth studies, especially on mSVF, focusing on its protein profile and the synergies between ASCs and accessorize cells whose molecular mechanisms could have a powerful clinical impact. While various biological aspects require further examinations, this research provided the first evidence of functional cell populations in the adipose tissue in mSVF similar to eSVF. Altogether, these findings showed phenotypic and functional similarities between the two adipose products and interesting viewpoints for the potential use of this mSVF as a minimally invasive approach also for OA care.

## Supplementary Information

ESM 1 (PDF 353 kb)
