# Supplementary material for: Prospects on the Potential In Vitro Regenerative Features of Mechanically Treated-Adipose Tissue for Osteoarthritis Care
Source: Stem Cell Rev Rep. 2021 Jan 19;17(4):1362–73. doi: 10.1007/s12015-020-10099-2 (PMC8316247; doi:10.1007/s12015-020-10099-2)
Supplement: Supplementary file 1 — (PDF 353 kb) [file 12015_2020_10099_MOESM1_ESM.pdf]

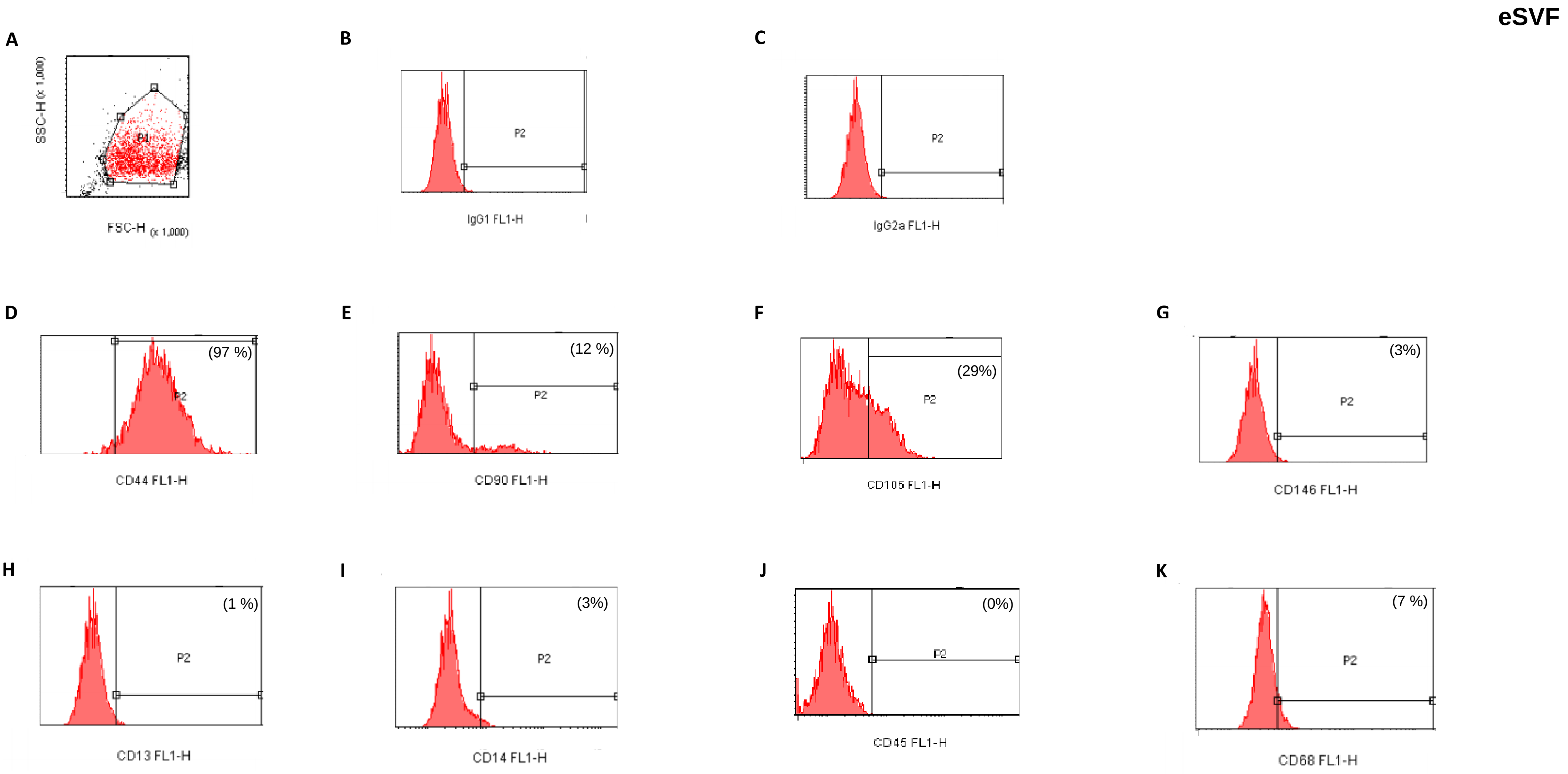

Supplementary Figure 1. A. The forward scatter (FSC)/ side scatter (SSC) gating graph in the eSVF group. Cells in the black circle represent the cell population analysed. B-C Histograms of isotype controls IgG1 and IgG2a. D-K. Histograms reporting the percentage of positivity (P2) of a representative specimen in the eSVF group for the following markers: CD-44, CD-90, CD-105, CD-146, CD-13, CD-14, CD-45 and CD-68.

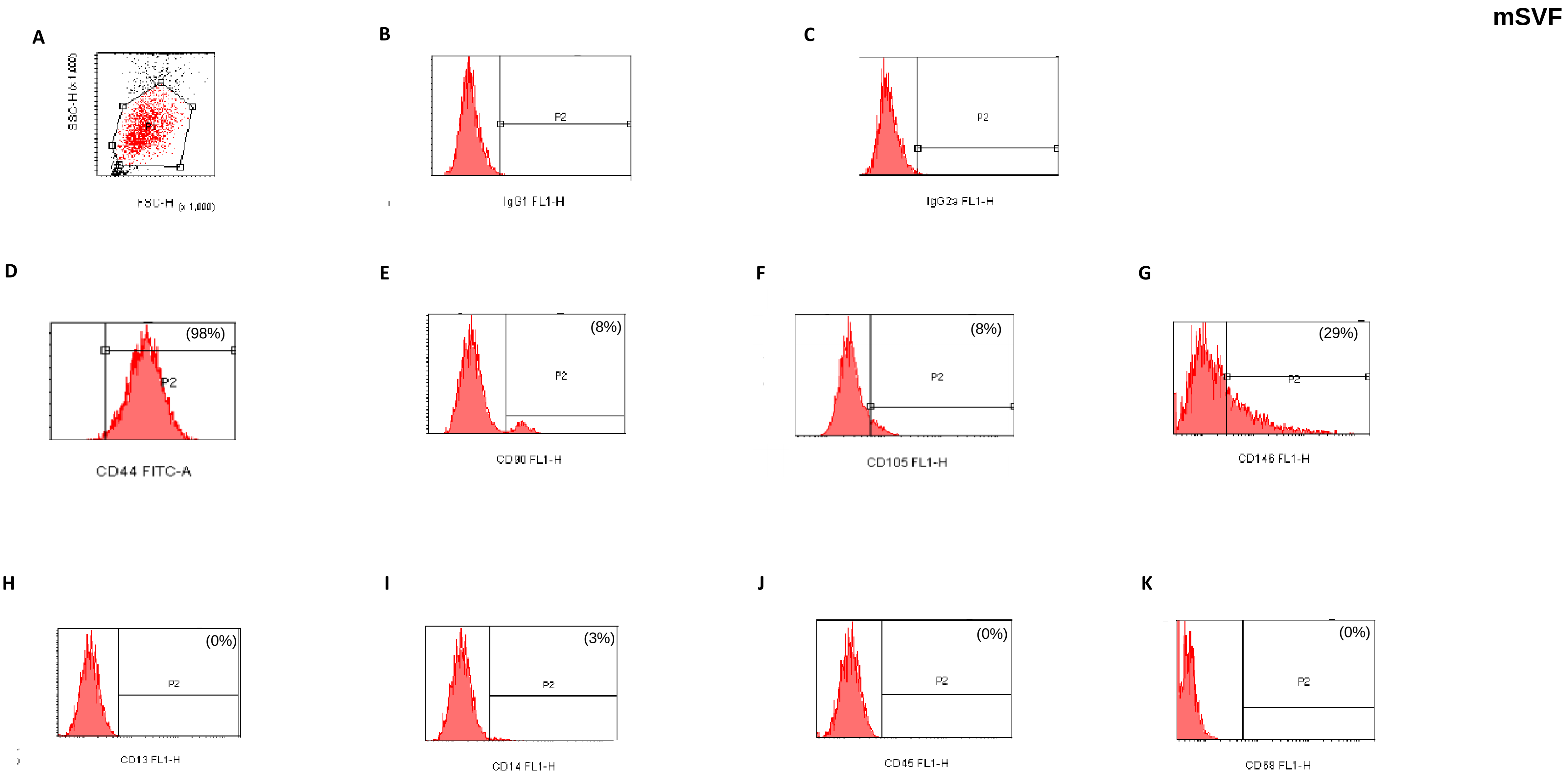

Supplementary Figure 2. A. The forward scatter (FSC)/ side scatter (SSC) gating graph in the mSVF group. Cells in the black circle represent the cell population analysed. B-C Histograms of isotype controls IgG1 and IgG2a. D-K. Histograms reporting the percentage of positivity (P2) of a representative specimen in the mSVF group for the following markers: CD-44, CD-90, CD-105, CD-146, CD-13, CD-14, CD-45 and CD-68.
